# COVID‐19, smoking, vaping and quitting: a representative population survey in England

**DOI:** 10.1111/add.15251

**Published:** 2020-09-28

**Authors:** Harry Tattan‐Birch, Olga Perski, Sarah Jackson, Lion Shahab, Robert West, Jamie Brown

**Affiliations:** ^1^ Institute of Epidemiology and Health Care University College London London UK; ^2^ SPECTRUM Consortium London UK

**Keywords:** Coronavirus, COVID‐19, e‐cigarettes, quitting, SARS‐CoV‐2, smoking, smoking cessation, vaping

## Abstract

**Aims:**

To estimate (1) associations between self‐reported COVID‐19, hand‐washing, smoking status, e‐cigarette use and nicotine replacement therapy (NRT) use and (2) the extent to which COVID‐19 has prompted smoking and vaping quit attempts and more smoking inside the home.

**Design:**

Cross‐sectional household surveys.

**Setting and participants:**

A representative sample of the population in England from April to May 2020. The sample included 3179 adults aged ≥ 18 years.

**Measurements:**

Participants who reported that they definitely or thought they had coronavirus were classified as having self‐reported COVID‐19. Participants were asked how often they wash their hands after returning home, before preparing foods, before eating or before touching their face. They were also asked whether, due to COVID‐19, they had (i) attempted to quit smoking, (ii) attempted to quit vaping and (iii) changed the amount they smoke inside the home.

**Findings:**

Odds of self‐reported COVID‐19 were significantly greater among current smokers [20.9%, adjusted odds ratio (aOR) = 1.34, 95% confidence interval (CI) = 1.04–1.73] and long‐term (> 1‐year) ex‐smokers (16.1%, aOR = 1.33, 95% CI = 1.05–1.68) compared with never smokers (14.5%). Recent (< 1‐year) ex‐smokers had non‐significantly greater odds of self‐reported COVID‐19 (22.2%, aOR = 1.50, 95% CI = 0.85–2.53). Bayes factors indicated there was sufficient evidence to rule out large differences in self‐reported COVID‐19 by NRT use and medium differences by e‐cigarette use. With the exception of hand‐washing before face‐touching, engagement in hand‐washing behaviours was high (> 85%), regardless of nicotine use. A minority (12.2%) of quit attempts in the past 3 months were reportedly triggered by COVID‐19, and approximately one in 10 current e‐cigarette users reported attempting to quit vaping because of COVID‐19.

**Conclusions:**

In England, current smokers and long‐term ex‐smokers appear to have higher odds of self‐reported COVID‐19 compared with never smokers in adjusted analyses, but there were no large differences between people who used nicotine replacement therapy or e‐cigarettes. Engagement in hand‐washing appears to be high, regardless of nicotine or tobacco use. A minority of past‐year smokers and current e‐cigarette users, respectively, report attempting to quit smoking/vaping due to COVID‐19.

## Introduction

COVID‐19 is a respiratory disease caused by the SARS‐CoV‐2 virus [1]. Tobacco smoking was considered an a priori risk factor for SARS‐CoV‐2 infection and poor COVID‐19 disease outcomes: current and former smoking is known to increase the risk of respiratory viral and bacterial infection and adverse disease outcomes compared with never smoking [2, 3]. Behavioural factors involved in both smoking and vaping, such as regular hand‐to‐mouth movements, may increase viral infection and transmission if performed without accompanying protective behaviours such as hand‐washing [4]. However, early descriptive epidemiology from the ongoing pandemic produced surprising results. Limited, mixed‐quality evidence suggests lower than expected smoking rates among those testing positive for SARS‐CoV‐2 infection and those hospitalized with COVID‐19 [5, 6]. This has led to the hypothesis that nicotine may protect against a hyperinflammatory response to SARS‐CoV‐2 infection [7, 8], thus preventing adverse outcomes such as hospitalization with COVID‐19 disease. Alternatively, the lower than expected smoking rates may reflect smokers being less likely to become infected due to an unexpected interaction between nicotine and ACE2 receptors, or may simply be an artefact of various measurement and sampling issues [5]. Our aim is to use a large, representative population survey with reliable assessment of smoking status to estimate rates of self‐reported SARS‐CoV‐2 infection in relation to smoking status.

The early literature has focused on smoking and COVID‐19 disease. However, comparisons of rates of COVID‐19 between smokers, ex‐smokers and non‐smokers are a poor test of the nicotine hypothesis. Other components in tobacco smoke could lead to greater symptom severity, which could obscure the hypothesized protective effect of nicotine. Electronic cigarettes (e‐cigarettes) and nicotine replacement therapy (NRT) have lower toxicity than cigarettes [9, 10]. Therefore, an assessment of the use of these products provides a better test of a potential protective effect of nicotine. Despite this, few studies so far in the pandemic have measured the use of e‐cigarettes or NRT [11]. In addition, most studies have not been able to rely upon representative sampling, and have failed to distinguish between current smokers, recent ex‐smokers, long‐term ex‐smokers and never smokers [5]. Our secondary aim is therefore to estimate rates of COVID‐19 by use of alternative nicotine products in a representative population survey.

Hand‐washing is an important behaviour in reducing the transmission of infectious diseases throughout the population, especially for those that are primarily spread through respiratory droplets [12]. Because of the repeated hand‐to‐mouth movements involved in smoking and vaping, current smokers and e‐cigarette users may be less likely to wash their hands regularly before touching their face. Additionally, personality factors, such as lower risk aversion [13], could lead to reduced hand‐washing among tobacco and nicotine users. These behavioural factors could explain differences in rates of COVID‐19 by smoking status, e‐cigarette use or NRT use. Therefore, in this study we measured associations between self‐reported engagement in hand‐washing behaviours and use of these products.

In the absence of clear evidence as to whether smoking is a risk factor for COVID‐19 or hospitalization, public health messaging has focused upon general benefits of quitting tobacco smoking [14, 15]. Some organizations, such as the American Lung Association [16], have also urged people to stop using e‐cigarettes (‘vaping’), claiming that doing so may reduce their risk of developing severe COVID‐19 symptoms—despite little evidence to support this. The COVID‐19 pandemic may therefore have prompted smokers and e‐cigarette users to attempt to stop in greater numbers.

Social distancing measures, introduced in England in March 2020, limited people to leave their home only for food, health care, exercise and essential work [17]. These measures may have increased the amount people smoke inside their home, which would be especially damaging because school closures meant that children were housebound and at risk of exposure to second‐hand smoke.

The COVID‐19 pandemic has exacerbated heath inequalities in England, with the highest mortality rates found among people working in manual, routine and service occupations [18] and in black and ethnic minority groups [19]. In addition, those living in deprived areas are the most likely to be diagnosed with COVID‐19 and have worse outcomes when hospitalized [20]. Because smoking and vaping prevalence is highest among people from disadvantaged groups [21], the impact these behaviours have on COVID‐19 may be more pronounced in these groups. In this study, we will use a representative population sample of adults in England to estimate:

1. associations between self‐reported COVID‐19 and smoking status, e‐cigarette use and NRT use;
2. associations between handwashing and smoking status, e‐cigarette use and NRT use;
3. the proportion of smokers and e‐cigarette users who have attempted to quit product use because they are motivated by the pandemic;
4. the proportion of smokers, with or without children in their household, who are smoking more, less, or the same amount inside their home; and
5. whether the above associations vary by socio‐economic status (SES).

## Methods

### Design

This study is part of the ongoing Smoking Toolkit Study (STS), a monthly cross‐sectional survey in England. It provides detailed information on smoking, vaping and NRT use. It recruits approximately 1700 participants per month using a combination of random location and quota sampling [22]. Interviews are performed with one household member until quotas based on factors influencing the probability of being at home (e.g. gender, age, working status) are fulfilled. Comparisons with sales data and other national surveys show that the STS recruits a representative sample of the population in England with regard to key demographic variables, smoking prevalence and cigarette consumption [22, 23]. Data are usually collected through face‐to‐face computer‐assisted interviews. However, social distancing restrictions under the COVID‐19 pandemic mean that data from April and May 2020 were collected via telephone, but relied upon the same combination of random location and quota sampling and weighting approach. Ethical approval was provided by the UCL Research Ethics Committee (0498/001).

### Study sample

Adults (≥ 18 years) who were interviewed in April and May 2020, the first survey waves to include questions concerning COVID‐19.

### Measures

#### Smoking status

Participants who reported currently smoking tobacco of any kind were considered current smokers. Those who reported having stopped smoking within the last year were considered recent ex‐smokers. Those who reported having stopped smoking more than a year ago were considered long‐term ex‐smokers. All others were considered never smokers. Never smokers were used as the reference category when calculating odds ratios (ORs). The term ‘past‐year smoker’ was be used to refer to the group of both current and recent ex‐smokers.

#### E‐cigarette and NRT use

Participants were asked which products they are currently using to cut down the amount they smoke, in situations when they are not allowed to smoke, to help them stop smoking, or for any other reason at all. Those who reported currently using e‐cigarettes were considered current e‐cigarette users and those who reported currently using NRT (e.g. gum, patches, lozenges) were considered current NRT users.

#### Self‐reported COVID‐19

Participants were told that the symptoms of coronavirus are high temperature/fever or a new, continuous cough. They were then asked which of the following statements best applies to them: (1) ‘I definitely have coronavirus’, (2) ‘I think I have coronavirus’, (3) ‘I definitely had coronavirus’, (4) ‘I think I had coronavirus’, (5) ‘I do not have or think I have had coronavirus’, (6) ‘don't know’ and (7) ‘prefer not to say’. Those who responded that they ‘definitely’ or ‘think they’ have/had coronavirus (1–4) were labelled as having a self‐reported COVID‐19.

#### Hand‐washing

Participants were asked how frequently, during the last week, they (i) washed their hands when they got home after being out, (ii) washed their hands before they ate, (iii) washed their hands before they prepared food and (iv) touched their eyes, nose or mouth without washing their hands first. For all questions, participants could respond ‘always’, ‘most of the time’, ‘about half the time’, ‘less than half the time’, ‘never’ or ‘don't know’. Responses were grouped into two categories: engaging in the behaviour the majority (most/always) of the time, coded 1, and all others, coded 0. For question (i), participants could respond that they have not left the house, and for question (ii), that they have not prepared food in the last week. Individuals who gave these responses were considered protected against infection, with responses coded as 1. Responses from question (iv) were reverse‐coded to reflect the protective behaviour of washing hands before touching eyes, nose or mouth.

#### Smoking quit attempt

Past‐year smokers who reported that they had made a quit attempt in the last 3 months were asked if their attempt was triggered by (i) the COVID‐19 outbreak or (ii) concern about future health problems.

#### Vaping quit attempt

Current e‐cigarette users were asked if they were trying to quit vaping completely because of the COVID‐19 outbreak.

#### Smoking inside the home

Smokers were asked whether, in the past month, they had been smoking more, the same amount or fewer cigarettes inside their home.

#### Children in household

Smokers were asked the number of children they had in their household. Responses were dichotomized as ≥ 1 and 0.

#### SES

Occupation‐based social grade [24] was used as our measure of SES, with responses dichotomized into ABC1 (managerial, professional and clerical workers) and C2DE (manual and casual workers, state pensioners and unemployed).

#### Potential confounders

In addition to SES, we included information on sex, age and region (London, North, Central, South)—variables probably associated with smoking status, e‐cigarette use, NRT use and COVID‐19 [18, 20, 25].

### Analysis

All analyses were conducted in R version 3.5 [26]. The pre‐registered analysis plan is available on the Open Science Framework (https://osf.io/vb9fm/). We made one amendment, which was to include data from May 2020 when these became available. Results following the original plan based on April 2020 data are available on‐line (https://osf.io/7ehx6/). Alpha was set to 5%. Where there were non‐significant results when assessing analysis 1 (A1), Bayes factors (BFs) were used to distinguish inconclusive data from evidence for no large, medium or small effect [27, 28]. Large effects in either direction were defined as having an OR = 4 or for lower estimates ¼, based on evidence that current smoking rates among patients hospitalized for COVID‐19 in China were four times lower than would be expected from the smoking prevalence in the country [6]. We defined a medium effect size as OR = 2 or ½ and a small effect size as OR = 1.5 or ⅔.

A1: Logistic regression was used to estimate the association between self‐reported COVID‐19 and (i) smoking status, (ii) e‐cigarette use and (iii) NRT use, with and without adjustment for potential confounders. We repeated the above analysis but included the interaction between SES and (i) to (iii) as an explanatory variable.
A2: Similarly, for each hand‐washing behaviour, we used logistic regression to estimate the association between engaging in the behaviour and (i) smoking status, (ii) e‐cigarette use and (iii) NRT use, with and without adjustment for potential confounders.
A3: We report the proportion of (i) past‐year smokers and (ii) e‐cigarette users who attempted to quit because of the outbreak, before and after stratifying by SES. For smokers, we also compared this to the proportion who attempted to quit because they were concerned about their future health.
A4: We also report the proportion of smokers, with or without children in their household, who were smoking more, less or the same amount inside their home. Logistic regression was used to estimate the association of smoking more (versus less/about the same amount) inside the home with children in household, SES and the interaction between SES and children in the household.

## Results

Of the 3285 participants interviewed, 3179 (96.8%) provided complete data on all questions required for the current analyses. Sample characteristics are shown in Table 1.

**Table 1:** Sample characteristics by self‐reported COVID‐19.

	Overall (*n* = 3179)	Self‐reported COVID‐19 (*n* = 510)	No self‐reported COVID‐19 (*n* = 2669)
Sex			
Women	1718 (54.0%)	266 (52.2%)	1452 (54.4%)
Age			
Mean (SD)	52.4 (17.6)	47.3 (17.6)	53.4 (17.6)
Region			
London	462 (14.5%)	101 (19.8%)	361 (13.5%)
North	938 (29.5%)	152 (29.8%)	786 (29.4%)
Central	951 (29.9%)	128 (25.1%)	823 (30.8%)
South	828 (26.0%)	129 (25.3%)	699 (26.2%)
SES			
ABC1	1969 (61.9%)	323 (63.3%)	1646 (61.7%)
C2DE	1210 (38.1%)	187 (36.7%)	1023 (38.3%)
Smoking status			
Never	1804 (56.7%)	261 (51.2%)	1543 (57.8%)
Long‐term ex	834 (26.2%)	135 (26.5%)	699 (26.2%)
Recent ex	72 (2.3%)	16 (3.1%)	56 (2.1%)
Current smoker	469 (14.8%)	98 (19.2%)	371 (13.9%)
E‐cigarette use			
No current use	2987 (94.0%)	468 (91.8%)	2519 (94.4%)
Current use	192^a^ (6.0%)	42 (8.2%)	150 (5.6%)
NRT use			
No current use	3091 (97.2%)	494 (96.9%)	2597 (97.3%)
Current use	88^a^ (2.8%)	16 (3.1%)	72 (2.7%)

^a^ Ninety‐three (48.4%) e‐cigarette users and 57 (64.8%) NRT users were current smokers. NRT = nicotine replacement therapy; SD = standard deviation; SES = socio‐economic status.

### A1: self‐reported COVID‐19

Of the sample, 16.0% [95% confidence interval (CI) = 14.8%–17.4%] reported a self‐reported COVID‐19. Table 2 shows the prevalence of self‐reported COVID‐19 by smoking status, e‐cigarette and NRT use. In adjusted analyses, current smokers and long‐term ex‐smokers had significantly greater odds of self‐reported COVID‐19 compared with never smokers. Current smokers also had significantly greater odds of self‐reported COVID‐19 than never smokers in unadjusted analyses. There were no other significant differences by smoking status, e‐cigarette use or NRT use, or significant interactions between product use and SES (Supporting information, Table S1). BFs indicated that there was insufficient evidence to rule out small, medium or large differences in self‐reported COVID‐19 between recent ex‐smokers and never smokers (Supporting information, Table S2). There was sufficient evidence to rule out (1) large unadjusted and adjusted associations with NRT use and (2) medium and large adjusted associations with e‐cigarette use.

**Table 2:** Associations of self‐reported COVID‐19 with smoking status, e‐cigarette use and NRT use.

Variable	*n* (%, 95% CI)	OR (95% CI)	*P*	aOR (95% CI)	*P*
Smoking status					
Never	261 (14.5%, 12.9–16.2%)	–	–	–	–
Long‐term ex	135 (16.1%, 13.8–18.8%)	1.18 (0.94–1.48)	0.16	1.33 (1.05–1.68)	0.02
Recent ex	16 (22.2%, 14.2–33.1%)	1.61 (0.92–2.69)	0.08	1.50 (0.85–2.53)	0.14
Current smoker	98 (20.9%, 17.5–24.8%)	1.44 (1.13–1.84)	< 0.01	1.34 (1.04–1.73)	0.02
E‐cigarette use					
No current use	468 (15.7%,14.4–17.0%)	–	–	–	–
Current use	42 (21.9%, 16.6–28.2%)	1.30 (0.91–1.81)	0.14	1.00 (0.68–1.43)	0.99
NRT use					
No current use	494 (16.0%, 14.7–17.3%)	–	–	–	–
Current use	16 (18.2%, 11.5–27.5%)	1.00 (0.55–1.68)	0.99	0.82 (0.44–1.41)	0.49

CI = confidence interval; NRT = nicotine replacement therapy; aOR = adjusted odds ratio. Adjusted analyses included terms for SES, sex, age and region. In addition, smoking status was added as a covariate when estimating associations between the outcome and e‐cigarette use and NRT use. Similar associations were found when (1) sample weights were not applied in adjusted analyses and (2) current smokers were excluded from the analysis of NRT and EC use (Supporting information, Table S3).

### A2: hand‐washing

Of participants, 95.6% (95.0–96.4%) reported washing their hands most of the time after coming home, 97.1% (96.5–97.6%) reported washing their hands most of the time before they prepared food, 87.2% (86.0–88.3%) reported washing their hands most of the time before they ate and 71.3% (69.7–72.9%) reported not touching their face without washing their hands at least half the time. Compared with never smokers, recent ex‐smokers had significantly lower odds of reporting washing their hands before touching their face the majority of the time, both before and after adjustment for potential confounding variables (see Table 3). All other associations of hand‐washing with smoking status, e‐cigarette use and NRT use were non‐significant.

**Table 3:** Associations between hand washing and smoking status, e‐cigarette use and NRT use.

Hand‐washing context	*n* (%, 95% CI)	unOR (95% CI)	*P*	aOR_j_ (95% CI)	*P*
After coming home					
Smoking status					
Never	1728 (95.8%, 94.8–96.6%)	–	*–*	–	*–*
Long‐term ex	803 (96.3%, 94.8–97.4%)	1.25 (0.82–1.94)	0.31	1.20 (0.78–1.89)	0.41
Recent ex	67 (93.1%, 84.8–97.0%)	0.67 (0.31–1.80)	0.37	0.76 (0.34–2.06)	0.55
Current smoker	446 (95.1%, 92.7–96.7%)	0.94 (0.61–1.48)	0.78	1.10 (0.71–1.76)	0.68
E‐cigarette use					
No current use	2859 (95.7%, 94.9–96.4%)	–	*–*	–	*–*
Current use	185 (96.4%, 92.7–98.2%)	1.61 (0.79–3.93)	0.24	1.87 (0.88–4.68)	0.14
NRT use					
No current use	2959 (95.7%, 95.0–96.4%)	–	*–*	–	*–*
Current use	85 (96.6%, 90.5–98.8%)	1.50 (0.55–6.15)	0.50	1.73 (0.62–7.25)	0.37
Before preparing food					
Smoking status					
Never	1758 (97.5%, 96.6–98.1%)	–	*–*	–	*–*
Long‐term ex	808 (96.9%, 95.5–97.9%)	0.66 (0.41–1.10)	0.10	0.71 (0.43–1.18)	0.18
Recent ex	69 (95.8%, 88.5–98.6%)	0.60 (0.22–2.40)	0.39	0.55 (0.20–2.23)	0.32
Current smoker	452 (96.4%, 94.3–97.7%)	0.67 (0.39–1.18)	0.15	0.65 (0.37–1.17)	0.14
E‐cigarette use					
No current use	2899 (97.1%, 96.4–97.6%)	–	*–*	–	*–*
Current use	188 (97.9%, 94.8–99.2%)	1.64 (0.67–5.48)	0.34	2.00 (0.79–6.83)	0.20
NRT use					
No current use	3002 (97.1%, 96.5–97.7%)	–	*–*	–	*–*
Current use	85 (96.6%, 90.5–98.8%)	1.11 (0.37–5.59)	0.87	1.40 (0.45–7.21)	0.62
Before eating					
Smoking status					
Never	1583 (87.7%, 86.2–89.2%)	–	*–*	–	*–*
Long‐term ex	720 (86.3%, 83.8–88.5%)	0.87 (0.68–1.12)	0.27	0.88 (0.68–1.14)	0.33
Recent ex	62 (86.1%, 76.3–92.3%)	1.03 (0.55–2.13)	0.94	0.94 (0.50–1.98)	0.87
Current smoker	407 (86.8%, 83.4–89.5%)	0.98 (0.74–1.32)	0.91	0.94 (0.70–1.28)	0.71
E‐cigarette use					
No current use	2604 (87.2%, 85.9–88.3%)	–	*–*	–	*–*
Current use	168 (87.5%, 82.1–91.5%)	1.20 (0.79–1.90)	0.42	1.22 (0.78–1.98)	0.40
NRT use					
No current use	2698 (87.3%, 86.1–88.4%)	–	*–*	–	*–*
Current use	74 (84.1%, 75.0–90.3%)	1.06 (0.59–2.09)	0.86	1.10 (0.59–2.21)	0.78
Before touching face					
Smoking status					
Never	1316 (72.9%, 70.9–74.9%)	–	*–*	–	*–*
Long‐term ex	576 (69.1%, 65.8–72.1%)	0.88 (0.73–1.06)	0.19	0.86 (0.71–1.04)	0.11
Recent ex	42 (58.3%, 46.8–69.0%)	0.55 (0.36–0.87)	< 0.01	0.57 (0.37–0.91)	.02
Current smoker	334 (71.2%, 67.0–75.1%)	0.89 (0.72–1.10)	0.28	0.94 (0.76–1.16)	0.54
E‐cigarette use					
No current use	2128 (71.2%, 69.6–72.8%)	–	*–*	–	*–*
Current use	140 (72.9%, 66.2–78.7%)	1.12 (0.83–1.53)	0.47	1.30 (0.94–1.81)	0.12
NRT use 0					
No current use	2204 (71.3%, 69.7–72.9%)	–	*–*	–	*–*
Current use	64 (72.7%, 62.6–80.9%)	1.02 (0.66–1.63)	0.92	1.12 (0.71–1.82)	0.63

CI = confidence interval; NRT = nicotine replacement therapy; aOR = adjusted odds ratio; unOR = unadjusted odds ratio. Adjusted analyses included terms for SES, sex, age and region. In addition, smoking status was added as a covariate when estimating associations between the outcome and e‐cigarette use and NRT use.

### A3: quitting

Table 4 shows that (i) COVID‐19 triggered smoking quit attempts among past‐year smokers who made a quit attempt in the past 3 months, (ii) COVID‐19 triggered vaping quit attempts among current e‐cigarette users and (iii) the above stratified by SES. There were similar rates of smoking and vaping quit attempts in advantaged and disadvantaged individuals; however, small sample sizes meant that there were large uncertainties in estimates.

**Table 4:** Smoking and vaping quit attempts segmented by SES.

Quit attempts	Overall, *N* (%, 95% CI)	ABC1, *n* (%, 95% CI)	C2DE, *n* (%, 95% CI)
Among past‐year smokers who attempted to quit in the last 3 months (*n* = 49)^a^			
Triggered by COVID‐19	6 (12.2%, 5.7–24.2%)	3 (13.0%, 4.5–32.1%)	3 (11.5%, 4.0–29.0%)
Triggered by future health concerns	20 (40.8%, 28.2–54.8%)	8 (34.8%, 18.8–55.1%)	12 (46.2%, 28.7–64.5%)
Among current e‐cigarette users (*n* = 170)			
Triggered by COVID‐19	19 (11.2%, 7.3–16.8%)	12 (12.6%, 7.4–20.8%)	7 (9.3%, 4.6–18.0%)

SES = socio‐economic status; CI = confidence interval.

^a^ Results among current smokers are shown in Supporting information, Table S4.

### A4: smoking inside the home

Of the 469 current smokers, 223 (47.5%, 43.1–52.1%) reported smoking the same number of cigarettes inside their home in the past month, 109 (23.2%, 19.6–27.3%) reported smoking fewer cigarettes inside their home, 112 (23.9%, 20.2–27.9%) reported smoking more cigarettes inside their home and 15 (3.2%, 1.9–5.2%) did not know. As is shown in Table 5, odds of smoking more inside the home did not significantly differ between advantaged and disadvantaged smokers or between those with or without children in the home. In addition, there was no significant interaction between SES and children in the home (OR = 1.79, 95% CI = 0.73–4.33, *P =* 0.20).

**Table 5:** Associations between smoking more inside the home, SES and children in the home.

Variable	*n* (%, 95% CI)	OR (95% CI)	*P*
SES			
ABC1	46 (20.4%, 15.7–26.2%)	–	–
C2DE	66 (27.0%, 21.9–32.9%)	1.42 (0.94–2.16)	0.10
Children in home			
Children	30 (20.5%, 14.8–27.8%)	–	–
No children	82 (25.4%, 20.9–30.4%)	1.29 (0.85–1.98)	0.23

SES = socio‐economic status; CI = confidence interval.

## Discussion

In this representative sample of adults in England, current smokers and long‐term ex‐smokers had significantly greater odds of self‐reported COVID‐19 compared with never smokers after controlling for a number of potential confounding variables. There was sufficient evidence to rule out a large association of self‐reported COVID‐19 with NRT use and a medium association with e‐cigarette use. Compared with never smokers, recent ex‐smokers had significantly lower odds of washing their hands before touching their face. Participants reported high engagement in other hand‐washing behaviours, regardless of smoking status, e‐cigarette use or NRT use. Of past‐year smokers who made a recent quit attempt, 12.2% were motivated to do so by COVID‐19 and a similar proportion (11.2%) of current e‐cigarette users attempted to quit vaping due to COVID‐19. Most current smokers reported smoking the same number of cigarettes inside their home, while equal proportions reported smoking more or fewer. No significant differences by SES were observed across analyses.

Prior studies unexpectedly found that smoking rates among those hospitalized for COVID‐19 were lower than would be expected from population estimates [5]. There are at least three possible explanations for the observation: smokers are less likely to become infected, smokers who are infected are less likely to develop disease severe enough to require hospitalization or it is an artefact of various measurement or sampling issues. In this study we found that, compared with never smokers, current smokers and long‐term ex‐smokers had greater odds of self‐reported COVID‐19. Recent ex‐smokers also had greater odds of self‐reported COVID‐19, but not significantly so. These findings suggest that one of the two explanations other than infection are likely to account for the lower than expected smoking rates among those hospitalized for COVID‐19. However, see below for an alternative interpretation.

In terms of infection, the most parsimonious interpretation of the current findings is that current smokers and long‐term ex‐smokers are at increased risk of SARS‐CoV‐2 infection, independent of a variety of socio‐demographic factors. This increased risk could be the result of behaviour or biology. A relevant behaviour is that smoking requires repeated hand‐to‐mouth actions, although we found no equivalent association with e‐cigarette use, which involves similar behaviours. In terms of biology, smoking has been associated with upregulation of ACE2, the receptor for the virus in the lung, which would provide a mechanism for increased risk. However, other studies argue the relationship between smoking and ACE2 is more complicated, and may vary between epithelial or alveolar cells [29, 30]. The alternative explanation is that smokers are more likely to self‐report COVID‐19, but that this does not reflect an increased infection risk. Instead, these differences could result from smokers and long‐term ex‐smokers having greater susceptibility to other infections with similar symptoms to COVID‐19 [3]. Consistent with this explanation is that current compared with never smoking is associated with reduced risk of testing positive among people tested in the community [5, 31]. In order to resolve these competing explanations for our observation that smokers had greater odds of reporting suspected COVID‐19, future studies need to assess the seroprevalence of SARS‐CoV‐2 infection by smoking status in large, representative, randomly selected samples of the population.

Beyond smoking, the current findings are also difficult to reconcile with nicotine being substantially protective against SARS‐CoV‐2 infection. Bayes factors indicated that there was sufficient evidence to rule out large differences in self‐reported COVID‐19 by NRT use and medium differences by e‐cigarette use. However, the current study was not able to address the possibility that nicotine may protect against the development of more severe COVID‐19 disease among people who become infected.

Protective behaviours, such as hand‐washing, are important in slowing the transmission of infections like SARS‐CoV‐2. Here we found that, regardless of smoking status or nicotine use, the majority of respondents (> 85%) reported that they wash their hands most of the time after coming home, before preparing food and before eating. Fewer people reported washing their hands most of the time before touching their face, and recent ex‐smokers had significantly lower odds than never smokers of reporting doing so. The primary route through which people become infected with SARS‐CoV‐2 is through cells in the lungs and mucus membranes of the eyes, nose and mouth [32]. Avoiding face‐touching without first washing one's hands could therefore be an important and understudied behaviour to reduce transmission [12].

Public health bodies have advised smokers that quitting may reduce their risk of severe COVID‐19 outcomes [15]. This advice is based on the observation that despite being hospitalized at lower than expected rates, current smoking is associated with greater disease severity among those who are hospitalized [5]. This advice has been shared through the news and in social media campaigns, such as ‘#QuitForCovid’ in the United Kingdom [14, 33]. Here, we found that, while COVID‐19 triggered quit attempts among some smokers in England, far more attempts were triggered by other reasons such as future health concerns. This is consistent with recently published results showing no increase in (i) internet searches for quitting smoking [34] or (ii) downloads of a popular smoking cessation application in the United Kingdom [35] following COVID‐19. It is possible that conflicting news stories, reporting the possibility that smoking is protective against COVID‐19 [36, 37], may have partially undermined attempts to encourage people to quit smoking. Some organizations have also advised people to stop vaping, arguing that e‐cigarette use makes one susceptible to SARS‐CoV‐2 infection—despite little evidence to support this [5, 16]. In our sample, one in 10 current e‐cigarette users attempted to quit vaping due to concerns about COVID‐19. These attempts to quit vaping might stem from the deteriorating perceptions about the harms associated with e‐cigarette use among smokers in England [38, 39].

There was a concern that social distancing measures might increase the amount people smoke inside their home, especially for disadvantaged individuals who may live in housing without easy access to outdoor smoking areas. Reassuringly, we found that the majority of participants reported smoking the same number of cigarettes inside the home, and equal numbers reported smoking more or fewer cigarettes. There was no significant association between smoking more inside the home and SES or children in the household. However, given our small sample and the wide confidence intervals, we may have lacked sufficient data to detect differences.

This study benefits from using a representative sample of the population in England, including questions about current e‐cigarette and NRT use, and distinguishing between never, current, recent ex‐smokers and long‐term ex‐smokers. However, there were a number of limitations. First, for some comparisons, sample sizes were small, which meant that there was substantial uncertainly in estimates. Secondly, our measure of COVID‐19 was self‐reported, and not confirmed with a viral or antibody test. Given that many other infections share symptoms with COVID‐19, some participants may have misdiagnosed themselves. In addition, it is likely that most participants with asymptomatic cases did not report being infected. Thirdly, individuals with the most severe cases of COVID‐19 were probably missing from our analysis, because they may have died or been hospitalized. Thus, if nicotine reduces the risk of severe symptoms, fewer infected nicotine users would be missing from the analysis than non‐users, leading to an upward bias in the OR between these groups. However, at its peak, only 0.02% of the UK population were in hospital with COVID‐19 [40]. This means that this sampling bias probably had a negligible effect on the estimated proportion of participants with self‐reported COVID‐19, which ranged from 14.5% of never smokers to 22.2% of recent ex‐smokers. Fourthly, social desirability bias may have led participants to over‐report their engagement in hand‐washing in order to be viewed favourably by their interviewer. Fifthly, because reported engagement in hand‐washing was so high, there may have been a ceiling effect whereby we were unable to detect differences by smoking status, e‐cigarette use or NRT use. Sixthly, only current e‐cigarette users were asked whether they had attempted to quit vaping because of concerns about COVID‐19, which meant that we did not capture those who had already quit vaping. Finally, there was no a priori sample size calculation, but we mitigated against this by calculating Bayes factors for nonsignificant results.

In conclusion, these results do not support the hypothesis that smoking or nicotine is protective against COVID‐19, with current smokers and long‐term ex‐smokers having significantly greater odds of self‐reported COVID‐19 than never smokers in adjusted analyses and no significant difference in odds of self‐reported COVID‐19 by use of e‐cigarettes or NRT. With the exception of washing hands before touching one's face, there was high engagement in hand‐washing irrespective of nicotine product use. Approximately one in 10 current e‐cigarette users reported attempting to quit vaping due to COVID‐19. As countries begin to relax social distancing measures, it is important to continue tracking how nicotine product use interacts with COVID‐19, and how this pandemic will influence smoking, vaping and quitting behaviours in the population.

## Declaration of interests

J.B. has received unrestricted research funding from Pfizer, who manufacture smoking cessation medications. L.S. has received honoraria for talks, an unrestricted research grant and travel expenses to attend meetings and workshops from Pfizer, and has acted as paid reviewer for grant‐awarding bodies and as a paid consultant for health‐care companies. R.W. has undertaken consultancy for companies that manufacture smoking cessation medications (Pfizer, J&J and GSK). All authors declare no financial links with tobacco companies or e‐cigarette manufacturers or their representatives.

## Author contributions

**Harry Tattan‐Birch**: Conceptualization; investigation; methodology; formal analysis; writing ‐ original draft. **Olga Perski:** Conceptualization; investigation; methodology; Writing ‐ review & editing. **Sarah Jackson:** Conceptualization; investigation; methodology; supervision; Writing ‐ review & editing. **Lion Shahab:** Conceptualization; investigation; methodology; Writing ‐ review & editing. **Robert West:** Conceptualization; investigation; methodology; Writing ‐ review & editing. **Jamie Brown:** Conceptualization; investigation; methodology; Writing ‐ review & editing.

## Supporting information

**Table S1** Interaction between SES and smoking status, e‐cigarette use and NRT use, when estimating suspected SARS‐CoV‐2 infection.
**Table S2** Bayes factors for non‐significant associations between suspected SARS‐CoV‐2 infection and smoking status, e‐cigarette use, and NRT use.
**Table S3** Sensitivity analysis estimating the association of suspected SARS‐CoV‐2 infection with e‐cigarette use and NRT use among individuals who were not current smokers.
**Table S4** Smoking quit attempts segmented by SES.

## References

[1] Li Q., Guan X., Wu P., Wang X., Zhou L., Tong Y., *et al*. Early transmission dynamics in Wuhan, China, of novel coronavirus–infected pneumonia. N Engl J Med 2020; 382: 1199–1207. 31995857 10.1056/NEJMoa2001316 PMC7121484

[2] Denholm J. T., Gordon C. L., Johnson P. D., Hewagama S. S., Stuart R. L., Aboltins C., *et al*. Hospitalised adult patients with pandemic (H1N1) 2009 influenza in Melbourne. Australia. Med J Aust 2010; 192: 84–86. 20078408 10.5694/j.1326-5377.2010.tb03424.x

[3] Feldman C., Anderson R. Cigarette smoking and mechanisms of susceptibility to infections of the respiratory tract and other organ systems. J Infect 2013; 67: 169–184. 23707875 10.1016/j.jinf.2013.05.004

[4] Simons D, Perski O, Brown J. Covid‐19: The role of smoking cessation during respiratory virus epidemics. BMJ News. Available at: https://blogs.bmj.com/bmj/2020/03/20/covid-19-the-role-of-smoking-cessation-during-respiratory-virus-epidemics/ (accessed 12 May 2020).

[5] Simons D, Shahab L, Brown J, Perski O. The association of smoking status with SARS‐CoV‐2 infection, hospitalisation and mortality from COVID‐19: a living rapid evidence review. Qeios, May 2020. 10.32388/UJR2AW.3 PMC7590402 33007104

[6] Farsalinos K, Barbouni A, Niaura R. Smoking, vaping and hospitalization for COVID‐19. Qeios, April 2020. 10.32388/z69o8a.13

[7] Farsalinos K., Niaura R., Le Houezec J., Barbouni A., Tsatsakis D., Vantarakis A., *et al*. Editorial: Nicotine and SARS‐CoV‐2: COVID‐19 may be a disease of the nicotinic cholinergic system. Toxicol Rep 2020; 7: 658–663. 32355638 10.1016/j.toxrep.2020.04.012 PMC7192087

[8] Kloc M., Ghobrial R. M., Kubiak J. Z. How nicotine can inhibit cytokine storm in the lungs and prevent or lessen the severity of COVID‐19 infection? Immunol Lett 2020; 224: 28–29. 32522666 10.1016/j.imlet.2020.06.002 PMC7836994

[9] Eaton D. L., Kwan L. Y., Stratton K., editors. Public Health Consequences of E‐Cigarettes. Washington, DC: US National Academies of Sciences; 2018: pp. 55–88. 29894118

[10] Shahab L., Goniewicz M. L., Blount B. C., Brown J., McNeill A., Alwis K. U., *et al*. Nicotine, carcinogen, and toxin exposure in long‐term E‐cigarette and nicotine replacement therapy users. Ann Intern Med 2017; 166: 390–400. 28166548 10.7326/M16-1107 PMC5362067

[11] Miyara M, Tubach F, Pourcher V, Morelot‐Panzini C, Pernet J, Horoche J*et al*. Low rate of daily active tobacco smoking in patients with symptomatic COVID‐19. Qeios, May 2020. 10.32388/WPP19W.4

[12] West R., Michie S., Rubin G. J., Amlôt R. Applying principles of behaviour change to reduce SARS‐CoV‐2 transmission. Nat Hum Behav 2020; 4: 1–9. 32377018 10.1038/s41562-020-0887-9

[13] Jusot F., Khlat M. The role of time and risk preferences in smoking inequalities: a population‐based study. Addict Behav 2013; 38: 2167–2173. 23454881 10.1016/j.addbeh.2012.12.011

[14] Today is the Day . Avalable at: https://www.todayistheday.co.uk/ (accessed 12 May 2020).

[15] Public Health England . Smokers at greater risk of severe respiratory disease from COVID‐19 ‐ GOV.UK. Gov.uk News. Available at: https://www.gov.uk/government/news/smokers-at-greater-risk-of-severe-respiratory-disease-from-covid-19 (accessed 8 May 2020).

[16] Rizzo A. What You Need to Know About Smoking, Vaping and COVID‐19. American Lung Association. Available at: https://www.lung.org/blog/smoking-and-covid19 (accessed 14 May 2020).

[17] UK Government . Coronavirus (COVID‐19): what you need to do—GOV.UK. Available at: https://www.gov.uk/coronavirus (accessed 8 May 2020).

[18] Windsor‐Shellard B, Kaur J. Coronavirus (COVID‐19) Related Deaths by Occupation*,* England and Wales—Office for National Statistics (ONS). London, UK: ONS; 2020. Available at: https://www.ons.gov.uk/peoplepopulationandcommunity/healthandsocialcare/causesofdeath/bulletins/coronaviruscovid19relateddeathsbyoccupationenglandandwales/deathsregistereduptoandincluding20april2020#women-and-coronavirus-related-deaths-by-occupation (accessed 21 June 2020).

[19] Aldridge R. W., Lewer D., Katikireddi S. V., Mathur R., Pathak N., Burns R., *et al*. Black, Asian and minority ethnic groups in England are at increased risk of death from COVID‐19: indirect standardisation of NHS mortality data. Wellcome Open Res 2020; 5: 88. 32613083 10.12688/wellcomeopenres.15922.1 PMC7317462

[20] Public Health England (PHE) . Disparities in the Risk and Outcomes from COVID‐19. London, UK: PHE; 2020. Available at: https://assets.publishing.service.gov.uk/government/uploads/system/uploads/attachment_data/file/892085/disparities_review.pdf (accessed 21 June 2020).

[21] McNeill A, Brose LS, Calder R, Bauld L, Robson D. Vaping in England: 2020 Evidence Update Summary. London, UK; 2020. Available at: https://www.gov.uk/government/publications/vaping-in-england-evidence-update-march-2020/vaping-in-england-2020-evidence-update-summary#vaping-among-adults (accessed 21 June 2020).

[22] Fidler J. A., Shahab L., West O., Jarvis M. J., McEwen A., Stapleton J. A., *et al*. ‘The smoking toolkit study’: a national study of smoking and smoking cessation in England. BMC Public Health 2011; 11: 479. 21682915 10.1186/1471-2458-11-479 PMC3145589

[23] Jackson S. E., Beard E., Kujawski B., Sunyer E., Michie S., Shahab L., *et al*. Comparison of trends in self‐reported cigarette consumption and sales in England, 2011 to 2018. JAMA Netw Open 2019; 2: e1910161. 31461148 10.1001/jamanetworkopen.2019.10161 PMC6716287

[24] Social Grade in National Readership Survey . Available at: http://www.nrs.co.uk/nrs-print/lifestyle-and-classification-data/social-grade/ (accessed 7 November 2019).

[25] Kock L., Shahab L., West R., Brown J. E‐cigarette use in England 2014–17 as a function of socio‐economic profile. Addiction 2019; 114: 294–303. 30306714 10.1111/add.14446 PMC6330091

[26] R Development Core Team . R: A Language and Environment for Statistical Computing. Vienna, Austria: R Foundation for Statistical Computing; 2008.

[27] Dienes Z. Using Bayes to get the most out of non‐significant results. Front Psychol 2014; 5: 781. 25120503 10.3389/fpsyg.2014.00781 PMC4114196

[28] Lakens D., McLatchie N., Isager P. M., Scheel A. M., Dienes Z. Improving inferences about null effects with Bayes factors and equivalence tests. J Gerontol Ser B 2018; 75: 45–57. 10.1093/geronb/gby065 29878211

[29] Oakes J. M., Fuchs R. M., Gardner J. D., Lazartigues E., Yue X. Nicotine and the renin‐angiotensin system. Am J Physiol Regul Integr Comp Physiol 2018; 315: R895–R906. 30088946 10.1152/ajpregu.00099.2018 PMC6295500

[30] Muus C., Luecken M. D., Eraslan G., Waghray A., Helmberg G., Sikkema L., *et al*. Integrated analyses of single‐cell atlases reveal age, gender, and smoking status associations with cell type‐specific expression of mediators of SARS‐CoV‐2 viral entry and highlights inflammatory programs in putative target cells. bioRxiv 2020; 10.1101/2020.04.19.049254

[31] Hopkinson N. S., Rossi N. N., Moustafa J. E., Laverty A. A., Quint J. K., Freydin M. B., *et al*. Current tobacco smoking and risk from COVID‐19 results from a population symptom app in over 2.4 million people. medrxiv 2020; 44; 10.1101/2020.05.18.20105288 33402392

[32] Lake M. A. What we know so far: COVID‐19 current clinical knowledge and research. Clin Med J R Coll Physicians London 2020; 20: 124–127. 10.7861/clinmed.2019-coron PMC7081812 32139372

[33] Prior R. Qutting smoking could help you fight Covid‐19. CNN. Available at: https://edition.cnn.com/2020/04/03/health/coronavirus-quitting-smoking-wellness/index.html (accessed 8 May 2020).

[34] Heerfordt C., Heerfordt I. M. Has there been an increased interest in smoking cessation during the first months of the COVID‐19 pandemic? A Google trends study. Public Health 2020; 183: 6–7. 32388011 10.1016/j.puhe.2020.04.012 PMC7167577

[35] Perski O., Herbeć A., Shahab L., Brown J. Influence of the SARS‐CoV‐2 outbreak on the uptake of a popular smoking cessation app in UK smokers: interrupted time series analysis. JMIR Mhealth Uhealth 2020; 8: e19494. 32463375 10.2196/19494 PMC7296974

[36] Chalmers V. More evidence smokers are at less risk of Covid‐19: Adults are half as likely to test positive. Daily Mail. Available at: https://www.dailymail.co.uk/news/article-8380289/More-evidence-smokers-risk-Covid-19-Adults-half-likely-test-positive.html (accessed 19 June 2020).

[37] Samuel H. Smokers ‘four times less likely’ to contract Covid‐19, prompting nicotine patch trials on patients. Telegraph. Available at: https://www.telegraph.co.uk/news/2020/04/23/smokers-four-times-less-likely-contract-covid-19-prompting-nicotine/ (accessed 19 June 2020).

[38] Tattan‐Birch H., Brown J., Shahab L., Jackson S. E. Association of the US outbreak of vaping‐associated lung injury with perceived harm of e‐cigarettes compared with cigarettes. JAMA Netw Open 2020; 3: e206981. 32539148 10.1001/jamanetworkopen.2020.6981 PMC7296387

[39] Perski O., Beard E., Brown J. Association between changes in harm perceptions and e‐cigarette use among current tobacco smokers in England: a time series analysis. BMC Med 2020; 18: 98. 32370755 10.1186/s12916-020-01565-2 PMC7201665

[40] Heneghan C, Oke J. COVID‐19: Admissions to Hospital—Update. 2020. Available at: https://www.cebm.net/covid-19/covid-19-uk-hospital-admissions/ (accessed 26 June 2020).

