# Supplementary material for: COVID‐19, smoking, vaping and quitting: a representative population survey in England
Source: Addiction. 2020 Sep 28;116(5):1186–95. doi: 10.1111/add.15251 (PMC8436761; doi:10.1111/add.15251)
Supplement: Supplementary file 1 — Table S1 Interaction between SES and smoking status, e‐cigarette use and NRT use, when estimating suspected SARS‐CoV‐2 infection. Table S2 Bayes factors for non‐significant associations between suspected SARS‐CoV‐2 infection and smoking status, e‐cigarette use, and NRT use. Table S3 Sensitivity analysis estimating the association of suspected SARS‐CoV‐2 infection with e‐cigarette use and NRT use among individuals who were not current smokers. Table S4 Smoking quit attempts segmented by SES. [file ADD-116-1186-s001.docx]

**Supplementary Material**

Supplementary Table 1: Interaction between SES and smoking status, e-cigarette use and NRT use, when estimating suspected SARS-CoV-2 infection.

| Variable | OR (95% CI) | p | OR_adj_ (95% CI) | p |
| --- | --- | --- | --- | --- |
| Smoking status * SES |  |  |  |  |
| Long-term ex: C2DE | 1.11 (0.70-1.77) | .65 | 1.18 (0.74-1.89) | .48 |
| Recent ex: C2DE | 0.43 (0.14-1.26) | .13 | 0.48 (0.15-1.43) | .19 |
| Current smoker: C2DE | 0.75 (0.45-1.23) | .25 | 0.73 (0.44-1.21) | .22 |
| E-cigarette use * SES |  |  |  |  |
| Current use: C2DE | 0.68 (0.34-1.37) | .28 | 0.73 (0.36-1.48) | .39 |
| NRT use * SES |  |  |  |  |
| Current use: C2DE | 0.58 (0.18-1.77) | .34 | 0.51 (0.16-1.59) | .25 |

Supplementary Table 2: Bayes factors for non-significant associations between suspected SARS-CoV-2 infection and smoking status, e-cigarette use, and NRT use.

|  | BF_un_ large | BF_un_ medium | BF_un_ small | BF_adj_ large | BF_adj_ medium | BF_adj_  small |
| --- | --- | --- | --- | --- | --- | --- |
| Smoking status |  |  |  |  |  |  |
| Never | - | - | - | - | - | - |
| Long-term ex | - | - | - | - | - | - |
| Recent ex | 0.84 | 1.38 | 1.60 | 0.55 | 0.93 | 1.17 |
| Current smoker | - | - | - | - | - | - |
| E-cigarette use |  |  |  |  |  |  |
| No current use | - | - | - | - | - | - |
| Current use | 0.37 | 0.68 | 0.99 | 0.13 | 0.26 | 0.46 |
| NRT use |  |  |  |  |  |  |
| No current use | - | - | - | - | - | - |
| Current use | 0.20 | 0.38 | 0.57 | 0.26 | 0.48 | 0.68 |

The alternative hypothesis was modelled as a normal distribution centred on zero, with a standard deviation equal to the expected effect size. Large expected effect sizes were set as OR = 4 or 1/4, medium expected effect sizes as OR = 2 or 1/2, small expected effect sizes as OR = 1.5 or 3/2.

Supplementary Table 3: Sensitivity analysis estimating the association of suspected SARS-CoV-2 infection with e-cigarette use and NRT use among individuals who were not current smokers.

| Variable | OR (95% CI) | p | OR_adj_ (95% CI) | p |
| --- | --- | --- | --- | --- |
| E-Cigarette use |  |  |  |  |
| No current use | - | - | - | - |
| Current use | 1.28 (0.76-2.06) | .32 | 0.96 (0.56-1.59) | .88 |
| NRT use |  |  |  |  |
| No current use | - | - | - | - |
| Current use | 0.89 (0.27-2.25) | .82 | 0.77 (0.23-2.00) | .62 |

Adjusted analyses included terms for SES, sex, age and region. In addition, smoking status was added as a covariate when estimating associations between the outcome and e-cigarette use and NRT use

Supplementary Table 4. Smoking quit attempts segmented by SES.

|  | Overall,  N (% [95% CI]) | ABC1,  N (% [95% CI]) | C2DE,  N (% [95% CI]) |
| --- | --- | --- | --- |
| **Among current smokers who attempted to quit in the last 3 months (n = 37)** |  |  |  |
| Triggered by COVID-19 | 4 (10.8%  [4.3%-24.7%]) | 2 (10.5%  [2.9%-31.4%]) | 2 (11.1%  [3.1%-32.8%]) |
| Triggered by future health concerns | 16 (43.2%  [28.7%-59.0%]) | 7 (36.8%  [19.1%-59.0%]) | 9 (50.0% [29.0%-71.0%]) |
